# Quantification of different microplastic fibres discharged from textiles in machine wash and tumble drying

**DOI:** 10.1007/s11356-020-11988-2

**Published:** 2020-12-18

**Authors:** Niina Kärkkäinen, Markus Sillanpää

**Affiliations:** Laboratory Centre, Finnish Environment Institute, Mustialankatu 3, FIN-00790 Helsinki, Finland

**Keywords:** Microplastic, Polyester, Polyamide, Polyacryl, Washing machine, Tumble drier, Household, Emission

## Abstract

Microplastic fibres released in synthetic cloth washing have been shown to be a source of microplastics into the environment. The annual emission of polyester fibres from household washing machines has earlier been estimated to be 150,000 kg in a country with a population of 5.5 × 10^6^ (Finland). The objectives of this study were (1) to quantify the emissions of synthetic textile fibres discharged from five sequential machine washes (fibre number and length) and tumble dryings (fibre mass) and (2) to determine the collection efficiency of two commercial fibre traps. The synthetic fabrics were five types of polyester textiles, one polyamide and one polyacryl. The number of fibres released from the test fabrics in the first wash varied in the range from 1.0 × 10^5^ to 6.3 × 10^6^ kg^−1^. The fibre lengths showed that the fleece fabrics released, on average, longer fibres than the technical sports t-shirts. The mass of fibres ranged from 10 to 1700 mg/kg w/w in the first drying. Fibre emissions showed a decreasing trend both in sequential washes and dryings. The ratio of the fibre emissions in machine wash to tumble drying varied between the fabrics: the ratio was larger than one to polyester and polyamide technical t-shirts whereas it was much lower to the other tested textiles. GuppyFriend washing bag and Cora Ball trapped 39% and 10% of the polyester fibres discharged in washings, respectively.

## Introduction

Plastics are synthetic materials made from mixtures of organic polymers and additives. They are used in a wide range of applications due to their low cost, ease of manufacture and many advantageous properties. In the year 2018, the world plastic production reached 359 million tonnes (Plastics 2019), a number which is expected to still rise in the future. During the last decade, microplastics have been observed in different kinds of environments all over the world which has raised concern in both the scientific community and the public. Microplastics are often defined as small solid synthetic polymer particles with the largest dimension less than 5 mm and the smallest dimension equal to 1 μm. They may contain functional additives and possible residual impurities such as bisphenol A, phthalates, flame retardants and UV absorbers. Microplastics have a large surface-area-to-volume ratio that contributes to their ability to act as vectors for different kinds of hydrophobic substances in the environment (Lee et al. 2014). The small size of the microplastics also makes them available for ingestion by small organisms (Su et al. 2018; Alomar and Deudero 2017; Hurley et al. 2017; Setälä et al. 2014).

Microplastics have been observed in numerous environments like the marine (Cincinelli et al. 2017; van der Hal et al. 2017; Lusher et al. 2015), freshwater (Rodrigues et al. 2018; Fischer et al. 2016; Mani et al. 2015) and terrestrial (Scheurer and Bigalke 2018; Zhang et al. 2018; Sruthy and Ramasamy 2017) environments. Although the numerous sources of microplastics have been identified, the quantitative contributions, pathways to the different environment compartments, environmental fate and ecological effects of microplastics are still largely unknown (Wang et al. 2020). Some of the previously identified microplastic sources include car tyres, personal care products, and synthetic textiles (Galafassi et al. 2019). The latter one has been determined to be a major source of microplastic fibres originating from textile laundering (Henry et al. 2019). The shed fibres travel from a washing machine into domestic wastewater which then enters a wastewater treatment plant. In Finland alone, the emissions of the most common synthetic fibres, i.e. polyester, from household machine washing, has been estimated to be 150,000 kg per year (Sillanpää and Sainio 2017).

The earlier studies have shown that the majority (≥ 98%) of the microplastics are removed from the wastewater when secondary and/or tertiary treatment processes are being used (e.g. Lares et al. 2018; Carr et al. 2016; Michielssen et al. 2016; Murphy et al. 2016; Talvitie et al. 2017; Magnusson and Norén 2014). The removed microplastics end up into sewage sludge during wastewater cleaning processes. The sewage sludge can then be applied onto a soil where microplastics may adversely affect soil animals (Selonen et al. 2020).

While domestic washing of synthetic textiles has been determined to be a significant source of microplastics entering the environment, fibre release from textiles is not yet well understood. Among these studies, polyester has been the most researched textile material due to its prevalence in the clothing industry (Carmichael 2015). In addition, some studies have included other textile materials, such as polyamide (Cesa et al. 2020; Yang et al. 2019; Carney Almroth et al. 2018; Hartline et al. 2016), polyacryl (Cesa et al. 2020; Carney Almroth et al. 2018; Napper and Thompson 2016) and blends of these materials (Corami et al. 2020; Belzagui et al. 2019). Previous studies have investigated the effects of washing conditions on fibre release, such as the use of detergent (Cesa et al. 2020; Corami et al. 2020; Kelly et al. 2019; Yang et al. 2019; Zambrano et al. 2019; Carney Almroth et al. 2018; De Falco et al. 2018; Hernandez et al. 2017) and the effects of sequential washing (Cesa et al. 2020; Belzagui et al. 2019; De Falco et al. 2019; Kelly et al. 2019; Zambrano et al. 2019; Carney Almroth et al. 2018; Hernandez et al. 2017; Sillanpää and Sainio 2017; Pirc et al. 2016; Napper and Thompson 2016). Fibre emission mitigating technologies have been in the focus of research only in three previous scientific papers: McIlwraith et al. (2019) investigated the collection efficiencies of two commercially available fibre traps, Cora Ball laundry ball and Lint LUV-R filter, whereas Yang et al. (2019) and Cesa et al. (2020) studied collection efficiencies of built-in filters of different washing machines.

This study focuses on the emissions of different synthetic fibres and their reduction during laundering. Microscopic and gravimetric analyses were done the determination of the synthetic fibres released in machine washes and tumble dryings, respectively. The objectives of the study are (1) to quantify the fibre release from five sequential cycles of machine wash and tumble drying and (2) to determine the collection efficiency of two commercial fibre traps. This is the first study that has investigated the fibre release from different synthetic textile materials in sequential tumble dryings and the collection efficiency of Guppyfriend washing bag.

## Materials and methods

### Textiles

**Fig. 1:**
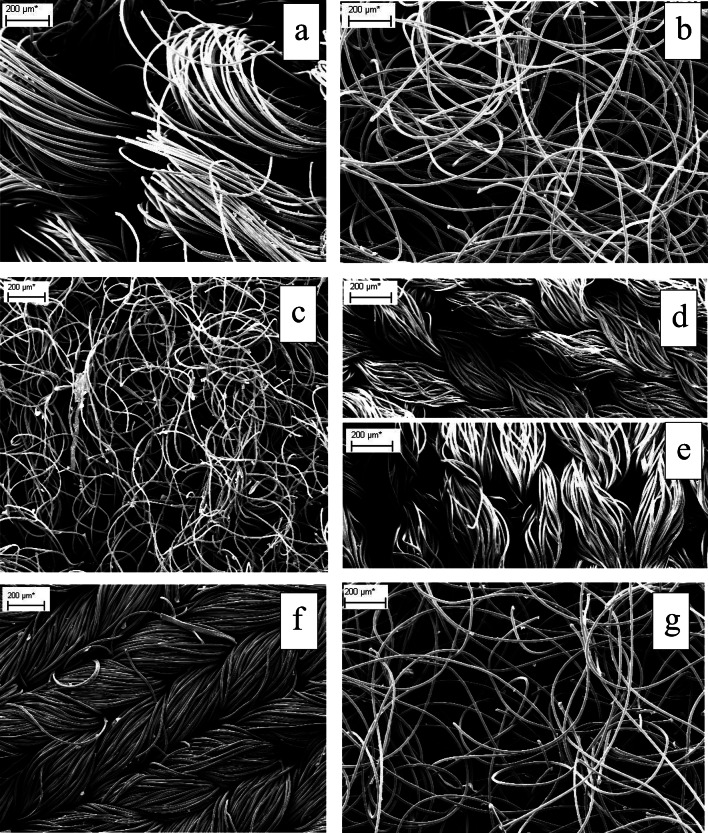
Scanning electron microscopy (SEM) images of the sample textiles, with × 50 magnification: **a** PES-fap; **b** PES-fnap; **c** PES-ss (the fleece layer); **d** PES-ts1; **e** PES-ts2; **f** PA-ts; **g** PAN-je

Five different types of polyester textiles, one type of polyamide t-shirt and one type of polyacryl jumper were selected for the study (Table 1): polyester anti-pill fleece fabric (PES-fap), polyester fleece blankets (PES-fnap), polyester softshell fabric (PES-ss), polyester technical sports t-shirts (PES-ts1 and PES-ts2), polyamide technical sport t-shirts (PA-ts) and acryl jumpers (PAN-je)**.** All the textiles studied were new and unused, with bright colours to differentiate them from each other. The textiles were also analysed with a scanning electron microscopy (ZEISS EVO 15) to the determination of fabric and yarn characteristics (Fig. 1). The sample textiles were sputter-coated with gold and analysed with a 20-kV electron beam. Finally, two different types of new and unused 100% polyester technical sports t-shirts (six dark grey and six green ones) were selected for the fibre trap experiments.

**Table 1:** Descriptions of the seven synthetic textiles used for washing and tumble drying

Sample	Description	Textile label	Area (m^2^)	Mass (g)	Thickness (mm)	Colour
PES-fap	One fabric, fleece, anti-pill	100% polyester	3.44	742.9	2.5	Red
PES-fnap	Two blankets, fleece, not anti-pill	100% polyester	5.00	679.4	0.15	Light blue
PES-ss	One softshell fabric	96% polyester, 4% elastane	3.37	1051.6	0.90	Orange and violet sides
PES-ts PES-ts1 PES-ts2	Four technical sport t-shirts	100% polyester	1.31 1.63	183.7 253.5	0.03 0.05	Two pink Two green
PA-ts	Four technical sport t-shirts (same shirts, different colours)	92% polyamide, 8% elastane	2.34	654.3	0.06	Two blue, two black
PAN-je	Two jumpers	100% acryl	1.64	575.1	–	olive green

### Machine washes and tumble drying

The synthetic textiles were washed with a front-load washing machine (Bosch WAE28477SN) using 50 ml of liquid detergent (pH = 8.0; Bio Luvil Color, Unilever). All the textiles were separately washed with the wash program ”Mix” with the settings as follows: water temperature 40 °C, spin-dry rate 1200 and total duration 75 min. Prior to each wash, the unloaded machine was cleaned with the liquid detergent by three times running the wash program “Super fast.” The settings of the wash programs have been detailed in Sillanpää and Sainio (2017). After each wash, the textiles were consequently dried in a front-load tumble drier (Bosch Serie 4). The program used for the tumble drying was “Timed Program Warm,” with the drying time of 50 min and with the “low heat” function turned on for a reduced temperature of 45 °C. Each textile went through five sequential washing-drying cycles.

The whole washing effluent was sampled into a large polyethylene barrel. Sub-sampling was done by taking three aliquots from the continuously stirred effluent water, with the volume varying from 50 to 1000 ml depending on the fibre concentration. The aliquots were then filtered through a gridded mixed-cellulose ester filter (diameter 47 mm, pore size 0.7 μm, type HC, Millipore, Bedford, MA, USA). The sub-sampling and filtration were done directly after each wash. The potential remaining fibres in the sub-sample containers were rinsed out with deionised water and then filtered. The wet filters were dried inside petri dishes with caps ajar in a laminar flow hood overnight. The sampling was described in detail by Sillanpää and Sainio (2017). In the tumble-drying experiments, the fibres were collected and rinsed off from the lint filter (mesh size 60 μm), then dried and stored in petri dishes until the gravimetric analysis.

### Quantification of microplastics

In the sequential washing experiments, the shed fibres were counted under an optical stereomicroscope (Nikon SMZ-1B, magnification × 35) from 10% of the total effective area of the filter. The resulting fibre numbers were converted to the fibre number in the whole effluent water. The determination of fibre number has been detailed in Sillanpää and Sainio (2017).

Gravimetric analysis with a microbalance (Mettler Toledo XP56) was performed for the fibres released in the tumble-drying experiments. The measurement of sample mass was done as a duplicate, and the resulting mean value was normalised in relation to the mass of the textile.

Fibre lengths in sequential washing were studied by measuring 50 fibres per textile in a fifth wash under a stereomicroscope (Olympus SZ61) equipped with a digital camera (Olympus SC50). Olympus cellSens Entry-imaging software (version 2.3) was used to manually measure fibre lengths. Magnification ranged from × 0.67 to × 4.5, depending on the fibre length.

### Fibre traps

**Fig. 2:**
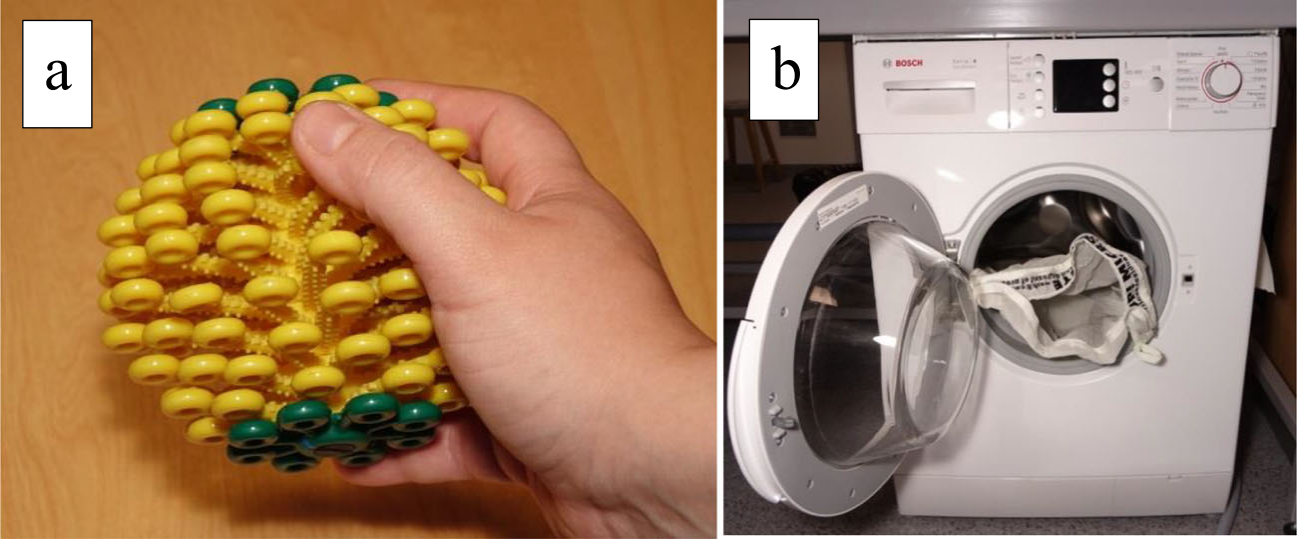
The photo of** a**) the Cora Ball and **b**) the Guppyfriend washing bag inside the washing machine

Two commercial fibre traps were tested with three replicates of 100 % polyester technical sports t-shirts. Guppyfriend washing bag (STOP! Micro Waste non-profit initiative) is a polyamide washing bag of size 45 × 68 cm, with a mesh size 30 μm (measured by the authors under an optical microscope). The bag is used by washing the laundry inside the bag and removing the trapped fibres by hand after washing. In addition to the efficiency, the Guppyfriend was also tested for how clean the textiles stained with blackcurrant juice and cream cheese got after washing inside the bag. Cora Ball (Rozalia Project) is a laundry ball made of soft, recycled plastic material. It is 13 cm in diameter (Fig. 2). The ball is placed inside the washing drum, where the Cora Ball’s narrow appendices trap fibres and piles. After washing, the stuck fibres are removed by hand. Both fibre traps have been designed to be easy-to-use and free of installation.

### Quality control

Sample contamination was minimised in all stages of experiments and analysis. The surfaces in contact with the samples were thoroughly cleaned with Milli-Q water prior to use. The white lab coat was worn while treating the samples. To prevent contamination from the fibres in the laboratory air, the filters were kept in closed petri dishes whenever possible. The only time when the filters were uncovered was during drying which took place in a laminar flow hood. As mentioned in the “Machine washes and tumble drying” section, the empty washing machine was washed with detergent after each wash. Possible fibre contamination originating from a previous wash was detected by alternating textiles of different colour. Three blank samples were taken from these intermediate washes to assess the level of contamination from stray fibres of the previous washes.

The uncertainties were estimated for three measurements described above. The measurement uncertainty based on the maximum relative standard deviation of the series of three replicate subsamples was 20 % for fibre number. For the mass measurement of fibre release in tumble dryings, the total uncertainty was assessed to be 25% due to the sampling uncertainty. The expanded measurement uncertainty (U) for fibre length was estimated to be 6 μm, i.e. ≤ 10% for the length range 55–565 μm, by using a Measurement Uncertainty kit (MUKit) software (Näykki et al. 2012).

## Results and discussion

### Fibre release in sequential machine washes

PES-ss shed the most fibres in the first wash (6.3 × 10^6^ kg^−1^), followed by the technical t-shirts PES-ts1 (3.1 × 10^6^ kg^−1^), PES-ts2 (1.4 × 10^6^ kg^−1^) and PA-ts (5.2 × 10^5^ kg^−1^). The fleece textiles shed 1.9 × 10^5^ kg^−1^ (PES-fnap) and 1.8 × 10^5^ kg^−1^ (PES-fap). The lowest emissions in the first wash were from PAN-je (1.0 × 10^5^ kg^−1^). As described in Table 2, the PES-ss fabric consists of two surface layers which are (1) woven fabric from 1.1dtex continuous filaments yarns (with elastane) and (2) a “fleece”-type fabric with piles generated by ripping of textured 0.8dtex filaments. Fibre release from the woven fabric is expected only from the seam and cut edges, whereas the breaking of individual piles from the fleece surface (or remains from the manufacturing) is an additional source of released fibres.

**Table 2:** Descriptions of the fabric and yarn characteristics of the textile samples

Sample	Description	Fabric	Yarn
PES-fap	Polar fleece fabric (100% polyester)	Single jersey knitted fabric with raised, looped piles on both sides,	Jersey: false-twist textured 3dtex multifilament yarn Piles: from flat (not-textured) 2dtex filaments
PES-fnap	Polar fleece blanket (100% polyester)	Single jersey knitted fabric with raised, looped piles on both sides	Jersey: false-twist textured 4dtex multifilament yarn Piles: from flat (not-textured) 2dtex filaments
PES-ss	Composite fabric: two layers laminated on a membrane film (1) Orange shell layer (2) Purple fleece layer (96% polyester, 4% elastane)	Shell: Plain weave fabric Fleece: Jersey knitted base fabric with raised, looped piles on one side	Plain weave: false-twist textured 1.1dtex PES multifilament yarn with embedded 15dtex elastane fibres in the chain and weft Jersey: false-twist textured 2.6dtex multifilament yarn Piles: from false-twist textured 0.8dtex filaments
PES-ts	Four technical sport t-shirts (100% polyester)		
PES-ts1^a^	Pink t-shirt	Single jersey knitted fabric	Stretch false-twist textured 0.9dtex multifilament yarn
PES-ts2^b^	Green t-shirt	Pique knitted fabric	Stretch false-twist textured 2dtex multifilament yarn
PA-ts^b^	Technical sport t-shirt (92 % polyamide, 8% elastane)	Single jersey knitted fabric	Stretch false-twist textured 1.5dtex multifilament PA yarn with elastane as rubber bands in the hip and shoulder
PAN-je	Knitted jumper (100% acryl)	Fluffy knitted (different knitting patterns)	3 brown and one black oval ~ 4.5dtex staple fibre yarn

^a^Seams were with cut edges but only partly covered with thread

^b^Seams were with cut edges and covered with thread

**Fig. 3:**
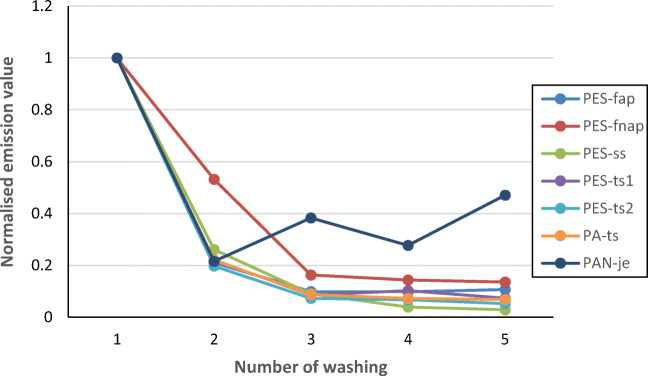
The normalised emission values of released synthetic fibres in five sequential washes. The normalisation was done by dividing the released fibre number of each wash by that of the first wash

The emissions of all textiles decreased in the sequential washes, with emission values in the fifth wash falling between 1.9 × 10^4^ and 1.9 × 10^5^ kg^−1^. Figure 3 shows the normalised emission values for the synthetic textiles tested. The initial emission values decreased in the following washes and the value was less than 20 % in the third wash except for PAN-je that had the normalised emission value between 0.22 and 0.47 in the second to fifth washes. The decreasing trends in the sequential washings have been earlier reported by Cesa et al. (2020), Belzagui et al. (2019), De Falco et al. (2019), Zambrano et al. (2019), Carney Almroth et al. (2018), Pirc et al. (2016) and Napper and Thompson (2016).

**Fig. 4:**
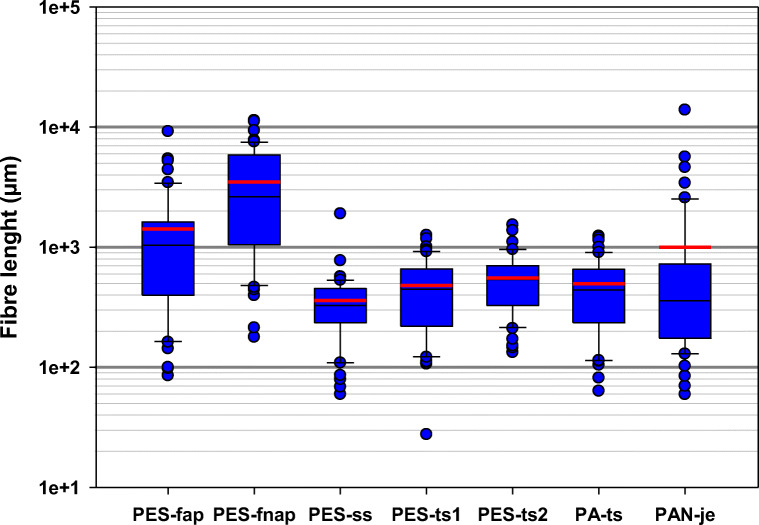
The length distribution of fibres released in the 5th washes (*n* = 50). The boundaries of the box indicate the 25th and 75th percentiles, the black line within the box signifies the median, the red line the mean, the whiskers the 10th and 90th percentiles, and the blue dots outlying points

The length distributions of fibres released in the 5th washes are shown in Fig. 4. The lengths varied greatly, from the shortest fibre length of 30 μm to the longest one of 14,000 μm. PES-fnap had the highest mean fibre length (3500 μm), followed by PES-fap (1400 μm). The mean fibre lengths of PES-ss and the technical t-shirts varied between 360 and 550 μm. The means were somewhat higher than the corresponding medians, with the exception of PAN-je which had the mean fibre length of 1000 μm but a clearly smaller median (360 μm). The high mean of PAN-je is accounted for a few exceptionally long acryl fibres (the longest 14,000 μm). In terms of length distribution range, technical sports t-shirts and PES-ss had a narrower range than the other textiles tested. It was noted that PAN-je had the most variation not only in length, but also in width. These variations in fibre lengths between the textiles can most likely be explained by their textile and yarn characteristics.

The fibre length from PAN-je ranged between 60 and 14,000 μm, and it had a mean length of 1000 μm. PAN-je is a knitted product, manufactured from bean-shaped staple fibres that typically have a length between 60 and 100 mm and a diameter of 18–28 μm. The fibres are pulled out of the fabric construction and broken during washing due to the mechanical stress.

The fibres that were released from the technical t-shirts PES-ts and PA-ts manufactured from continuous filaments were mostly short, with a mean length of 480 μm for PES-ts1, 550 μm for PES-ts2 and 500 μm for PA-ts. The fibre lengths for the t-shirts ranged between 30 and 1500 μm. The origin of these fibres was most likely both the cut edges of the fabric and the seams that contained damaged fibres produced during manufacturing when a needle pierced the fabric.

The fibre lengths from the polar fleece textiles might be explained by the looped pile heights of the fabric which is about half the length of the fibres forming the looped piles. The height of the piles on the one-sided fleece of the PES-ss fabric was about 1000 μm, and the released fibres had a mean length of 360 μm. The double-sided fleece PES-fap had piles with heights of 1000 μm and 2000 μm, with the released fibres having a mean length of 1400 μm. The pile heights of the double-sided fleece PES-fnap were 900 μm and 800 μm, but the measured mean fibre length was unexpectedly high (3500 μm). These long fibres could be explained by a poor embedment of the piles in the fabric which enables longer fibres to be pulled out.

Previous studies have focused either the complete textile products or textile samples cut from synthetic fabrics. Pirc et al. (2016) studied the washing of a double-sided fleece blanket with a pile height of 1000 μm. They reported higher fibre lengths for the released fibres when compared with the present study: a mean fibre length of 5300 μm and a wide fibre length distribution with lengths ranging from 300 to 25,000 μm which refers to slightly longer fibres than in the present study. Hernandez et al. (2017) studied the fibre length distributions of two textiles samples cut from knitted (single jersey and interlock) polyester fabrics during five simulated home sequential washings. In comparison with the present study, they used two different polyester fabrics knitted from yarns made from staple fibres. The cut edges were folded and sealed with a thread. Their fibre size distributions in fifth washing were similar to those of technical t-shirts in the present study.

The pore size of the filter used for the filtration of the washing effluent in the present study (0.7 μm) was close to those (0.2 to 0.7 μm) used by Corami et al. (2020) and Hernandez et al. (2017) but smaller than those (20 to 200 μm) used by Cesa et al. (2020), Belzagui et al. (2019), Kelly et al. (2019), De Falco et al. (2018), Hartline et al. (2016), Napper and Thompson (2016) and Pirc et al. (2016). In terms of fibre number per fabric mass, the smaller the filter pore size was, the higher the fibre emission values were reported. The highest fibre emissions were from the present study, Corami et al. (2020) and De Falco et al. (2018). All the mentioned studies had emission values reaching millions of fibres per textile mass (kg).

### Fibre release in sequential tumble dryings

**Fig. 5:**
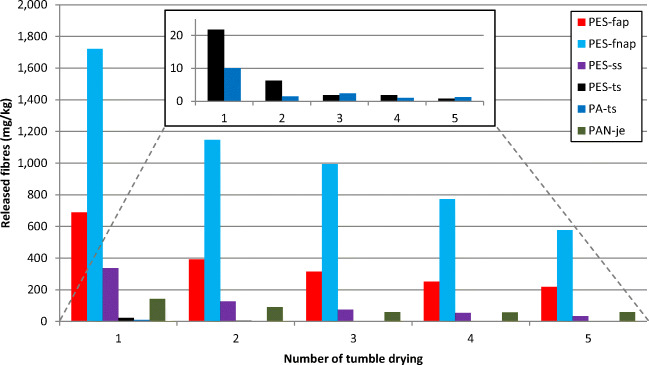
Fibre masses released from six different textiles in five sequential tumble dryings

As with the sequential washing, fibre emissions showed a decreasing trend also in sequential drying (Fig. 5), with some of the fibre emissions reaching a plateau within the five sequential dryings. The first tumble-drying released the most fibres in all the textiles: the highest emissions were from PES-fnap (1700 mg/kg), PES-fap (690 mg/kg) and PES-ss (340 mg/kg). All these three textiles were fleece or contained fleece that is the fabric with raised, looped piles. They were followed by the fluffy knitted PAN-je (140 mg/kg). The fabrics releasing the least fibres in the first drying were the PA-ts (10 mg/kg) and PES-ts (22 mg/kg, with PES-ts1 and PES-ts2 textiles tumble dried together). The double-sided fleece textiles PES-fnap and PES-fap continued to release considerably more fibres than other textiles, and without stabilizing their emissions, throughout the sequential dryings. The technical t-shirts PA-ts and PES-ts continued to release the least amount of fibres throughout the sequential dryings.

The high fibre emission values of PES-nfap, PES-fap and PES-ss during tumble drying (Fig. 5) result likely from the loosely knitted structure of the fabrics, i.e. that the fabrics have on their surface both raised fibre-ends and raised, looped piles. These kinds of loose fibres are susceptible to being broken off from the textile surface, for example due to mechanical stress from washing (Zambrano et al. 2019). In general, loosely knitted fibres are also easily entangled together to form pills on the textile surface (Hussain et al. 2008), for example during washing-drying cycles (Okubayashi and Bechtold 2005; Okubayashi et al. 2005). The formed pills can then be worn away from the textile surface due to mechanical stress. While fibre loss from pilling has been discussed in relation to machine wash (Napper and Thompson 2016), it should be also considered in relation to tumble drying.

PAN-je, manufactured from staple fibres, also has many raised fibre-ends (no piles) that should be susceptible to fibre loss during drying. This shows in the emission values of PAN-je in that they are placed between the other textiles. Finally, the technical t-shirts PA-ts and PES-ts, manufactured from continuous filaments, have “less hairy” and more firmly knitted textile surface without piles or multiple fibre-ends sticking out of the surface. This likely results in less fibre release than the other textile samples during tumble drying. The t-shirts only have fibre-ends sticking-out from their seams and from the cut edge of the fabric from which fibres might have been released during drying, as well as during washing.

**Fig. 6:**
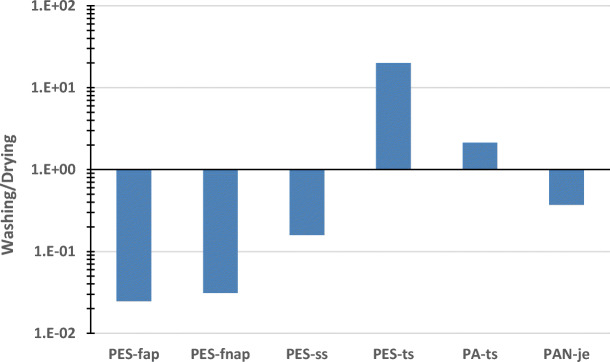
The machine wash-to-tumble drying ratio of the fibres released from the fifth treatment of the six textiles

For comparison of fibre emissions from washings and dryings, the fibre numbers from machine washes were converted to the fibre mass by multiplying the emission numbers by the fibre linear density (dtex values in Table 2) and the mean length of released fibres. The machine wash-to-tumble drying ratio of the fibres released from the fifth treatment is presented in Fig. 6. The ratios of polyester and polyamide technical t-shirts are higher than 1, which indicates the fibre release being larger in machine wash than in tumble drying. The other tested textiles had the ratio lower than 1, which refers the tumble drying to be the dominating treatment in fibre release.

Fibre release can be accounted for two different mechanisms: (1) the detachment of already loose fibres from the fabric surface, produced for example from the manufacturing process of the fabric, and (2) the breaking-off of fibres from the fabric itself. Tumble drying the textiles was accompanied by more mechanical stress than when washing the textiles in water. Therefore, the breaking-off of fibres from the fabric itself during tumble drying is expected to be higher than during washing. Some fibres released during washing were already loose on the fabric surface or weakened from previous treatments to break off. The t-shirts were more firmly knitted fabrics with fibre-ends sticking-out only in the seam and the cut edges of the fabric. Also, loose fibres might have been generated during the stitching of the seam. The loose fibres, broken off from continuous filaments by a needle, may have been released during washing with declining fibre numbers during the sequential wash-and-drying cycles.

Pirc et al. (2016) is the only study where fibre release during tumble drying has been investigated. They treated six 100% polyester fleece blankets in ten sequential wash-and-drying cycles. Like in the present study, they observed a decreasing trend in the fibre emissions. The fibre emissions in the first drying (washing done with liquid detergent) was 200 mg/kg which then decreased to 61 mg/kg in the fifth drying, and finally reaching 34 mg/kg in the tenth drying. For the first five dryings, they reported lower fibre emission values for fleece fabrics than the present study. The differences between these two studies are likely due to differences in fabric characteristics and drying conditions. The mesh size of the lint filter was larger (180 μm) in Pirc et al. (2016) than in the present study (60 μm) but also the drying program and the shape of the drying drum may have affected the fibre release. More research should be done on tumble drying to better understand the effects of different fabric characteristics and drying conditions on the fibre emissions during tumble drying.

It must be noted that the fibres released in the tumble drying will not be led straight into a wastewater treatment plant, unlike the fibres released in machine wash or washer dryers. Instead, the fibres are trapped in the tumble dryer’s lint filter that is cleaned by hand, with a vacuum cleaner or washing with water. Thus, it is up to the consumer whether the trapped fibres end up into the trash or sewage water. O’Brien et al. (2020) have shown that tumble drying releases microplastic fibres into indoor air, though the concentrations were low (1.6 ± 1.8 fibres/m^3^) in their study. It is worth mentioning that the residence time of large particles/fibres (much larger than 10 μm) is typically short in the air and therefore they deposit in the vicinity of their emission source.

### Efficiency of fibre traps

**Fig. 7:**
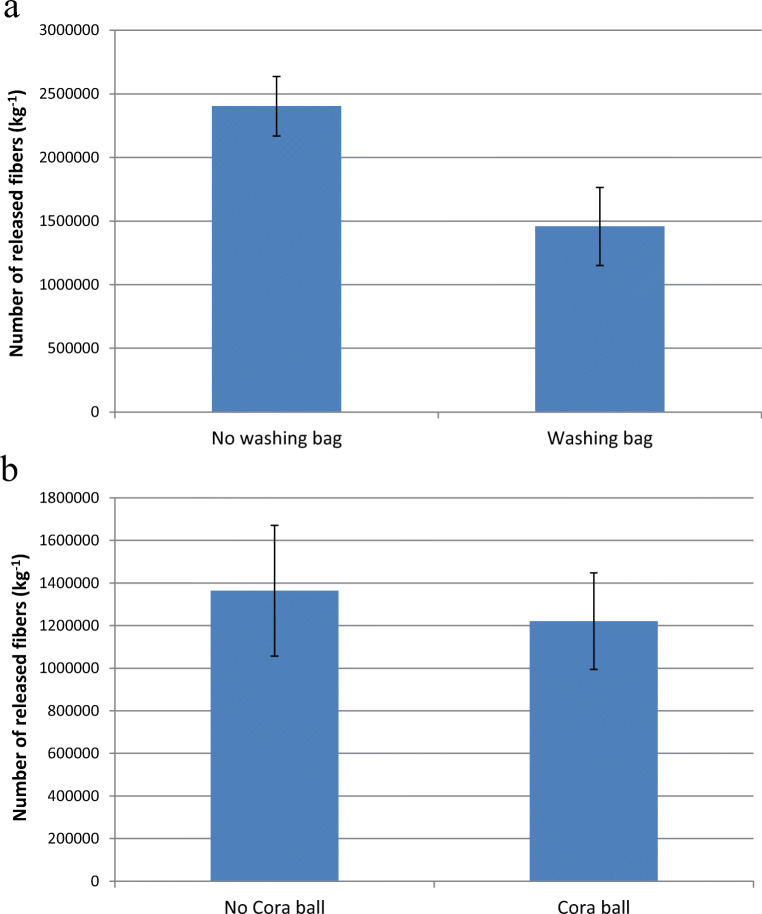
The number of the released polyester fibres with and without **a** the Guppyfriend washing bag and **b** the Cora Ball. Mean ± SD of three replicate samples

The collection efficiencies of two commercial fibre traps are presented in Fig. 7. The Guppyfriend clearly reduces fibre emissions during washing, with 39% reductions observed with fibre numbers. It should be noted that the collection efficiency is likely higher for the loosely knitted textiles that release longer fibres than the firmly knitted textiles studied here. The Guppyfriend did not prevent the stains of blackcurrant juice and cream cheese from being removed from the textile. Thus, the Guppyfriend can be used to reduce fibre emissions without compromising cleaning efficiency.

The Cora Ball trapped 10% of the short polyester fibres studied here (Fig. 7). McIlwraith et al. (2019) has also investigated the collection efficiency of Cora Ball. They found that Cora Ball mitigated fibre emissions by 26% on a basis of fibre number. The difference between the two studies can be explained by the different length of studied fibre (longer than 100 μm in McIlwraith et al. 2019), since the trapping efficiency of Cora Ball increases with the size (length and lint) of fibres.

The overall effectiveness of a fibre trap to mitigate microplastic pollution is greatly impacted by their user-friendliness. Herweyers et al. (2020) examined the perceptions and attitudes of customers toward products that mitigate fibre emissions from domestic washings. Based on questionnaires (*n* = 411) and user observations with interviews (*n* = 8), they found that the effectiveness and durability of a product followed by its usability, with special focus on convenience, were the most important factors in convincing people to use the product. They concluded that to keep people using the product for a long time, the product should be simple-to-use and user friendly. With both Cora Ball and Guppyfriend, the consumer needs to both clean the traps and dispose the collected fibres by hand. However, compared with other commercial fibre traps like the external Lint LUV-R filter, Cora Ball and Guppyfriend do not need to be installed or similarly maintained. There is also no danger of blockage when a filter is not changed often enough. Overall, there is a lower threshold for a consumer to purchase a Cora Ball or a Guppyfriend than an external filter to combat fibre emissions during washing.

## Summary and conclusions

This study presented the emissions of seven synthetic textile fibres discharged from five sequential machine washes and tumble dryings. In addition, two commercial fibre traps were tested for how effectively they can catch the fibres released during the machine wash. In the first wash, the number of released fibres ranged from 1.0 × 10^5^ to 6.3 × 10^6^ kg^−1^. The mass range in the first tumble drying was from 10 to 1700 mg/kg. Overall, the fibre emissions exhibited a decreasing trend in both sequential washes and dryings. The fibre lengths from sequential washing showed that the fleece fabrics released, on average, longer fibres than the technical sports t-shirts.

The GuppyFriend washing bag and the Cora Ball trapped 39% and 10% of the polyester fibres discharged in washings, respectively. Thus, both traps mitigate the emissions, they are easy-to-use and free of installation, but they are not as effective as consumers may assume. As in the case of fibres caught in tumble drying, it is up to the consumer whether the trapped fibres end up into the trash or sewage water. This shows that new technical solutions can decrease the fibre emissions, but the consumer choice, habits and education play also important roles.

## Data Availability

Not applicable.
